# Development of a tool for identifying and addressing prioritised determinants of quality improvement initiatives led by healthcare professionals: a mixed-methods study

**DOI:** 10.1186/s43058-020-00082-w

**Published:** 2020-10-23

**Authors:** Anne A. C. van Tuijl, Hub C. Wollersheim, Cornelia R. M. G. Fluit, Petra J. van Gurp, Hiske Calsbeek

**Affiliations:** 1 Department of IQ Healthcare, Radboud Institute for Health Sciences, Radboud University Medical Center, Geert Grooteplein 21, 6525 EZ Nijmegen, the Netherlands; 2 Department for Research in Learning and Education, Radboudumc Health Academy, Gerard van Swietenlaan 2, 6525 GB Nijmegen, the Netherlands; 3 Department of Internal Medicine, Radboud University Medical Center, Geert Grooteplein Zuid 10, 6525 GA Nijmegen, the Netherlands

**Keywords:** Determinants of practice, Quality improvement, Healthcare professionals, Tool development

## Abstract

**Background:**

Several frameworks have been developed to identify essential determinants for healthcare improvement. These frameworks aim to be comprehensive, leading to the creation of long lists of determinants that are not prioritised based on being experienced as most important. Furthermore, most existing frameworks do not describe the methods or actions used to identify and address the determinants, limiting their practical value. The aim of this study is to describe the development of a tool with prioritised facilitators and barriers supplemented with methods to identify and address each determinant. The tool can be used by those performing quality improvement initiatives in healthcare practice.

**Methods:**

A mixed-methods study design was used to develop the tool. First, an online survey was used to ask healthcare professionals about the determinants they experienced as most facilitating and most hindering during the performance of their quality improvement initiative. A priority score was calculated for every named determinant, and those with a priority score ≥ 20 were incorporated into the tool. Semi-structured interviews with implementation experts were performed to gain insight on how to analyse and address the determinants in our tool.

**Results:**

The 25 healthcare professionals in this study experienced 64 facilitators and 66 barriers when performing their improvement initiatives. Of these, 12 facilitators and nine barriers were incorporated into the tool. Sufficient support from management of the department was identified as the most important facilitator, while having limited time to perform the initiative was considered the most important barrier. The interviews with 16 experts in implementation science led to various inputs for identifying and addressing each determinant. Important themes included maintaining adequate communication with stakeholders, keeping the initiative at a manageable size, learning by doing and being able to influence determinants.

**Conclusions:**

This paper describes the development of a tool with prioritised determinants for performing quality improvement initiatives with suggestions for analysing and addressing these determinants. The tool is developed for those engaged in quality improvement initiatives in practice, so in this way it helps in bridging the research to practice gap of determinants frameworks. More research is needed to validate and develop the tool further.

Contributions to the literature

- In the existing literature, there are various determinant models to understand the influence of barriers and facilitators on implementation outcomes.
- Although the literature agrees to a large extent on which determinants are important, it is less clear which determinants are experienced as most important by those engaged in quality improvement in the field, and how to find methods to analyse and address these determinants.
- This study describes a tool of prioritised facilitators and barriers for healthcare quality improvement initiatives, with suggestions for analysing and addressing them. This tool contributes to bridging the research to practice gap by taking a serious look at how practical research-centric determinant frameworks are for those engaged in quality improvement initiatives in the field.

## Background

The importance of contextual determinants for the success of quality improvement (QI) initiatives is widely accepted [1, 2]. The increased interest in the context where QI initiatives are implemented has resulted in the publication of several frameworks of determinants in the literature (e.g. Tailored Implementation for Chronic Disease (TICD [2]), Consolidated Framework for Implementation Research (CFIR [3]), Model for Understanding Success in Quality (MUSIQ [4]) and Measurement Instrument for Determinants of Innovations (MIDI [5])). These frameworks describe general classes of determinants, which are independent variables that prevent or enable implementation outcomes, such as changes in healthcare professional behaviours [2, 6]. These determinants are also described as barriers and enablers, barriers and facilitators or problems and incentives [2]. The aim of these frameworks is to help implementation researchers and people responsible for QI initiatives in healthcare to identify determinants, enabling them to design and execute more effective implementation strategies to support implementation of the QI initiative [6].

In this article, we use QI to refer to improvements in healthcare related to more effective, safe, efficient, timely and patient-centred care [7]. QI initiatives are defined as, e.g. new procedures, technologies, guidelines, protocols and programmes that are firmly established as being able to contribute to more effective, safe, efficient, timely and patient-centred care [8]. With the term implementation, we refer to a planned process and systematic introduction of these initiatives; the aim being that these are given a structural place in professional practice, in the functioning of organisations or in the health care structure [7]. Implementation strategies in healthcare can be defined as methods or techniques used to enhance the adoption, implementation and sustainability of a clinical practice or programme [9].

Most of the existing generic frameworks of determinants aim to be comprehensive, including all domains and determinants [2]. These frameworks are based on the idea that implementation is a multidimensional phenomenon taking place in complex settings with multiple interacting influences within and across different types of determinants [6]. The downside is that these frameworks lead to long lists of determinants (e.g. TICD contains 57 determinants), which can be challenging to use in practice; it would be difficult to analyse and address all of these determinants during implementation [10]. For healthcare professionals planning to implement an improvement initiative, a shorter tool of determinants that are prioritised based on experienced importance by those engaged in QI in the field would be useful; however, to our knowledge, no such tool yet exists.

In most general determinant frameworks, it is unclear whether the barriers and facilitators are the most important determinants within a specific context and for a specific population. A systematic review of 12 available frameworks and taxonomies of determinants of practice revealed that most were based on literature reviews or developed using brainstorming and consensus processes [2]. A recent scoping review [11] of determinant frameworks showed that many were developed based on earlier frameworks, which could lead to a narrow approach to exploring and understanding determinants. Although some frameworks are based on the author’s own implementation experiences [11], it is unclear to what extent these determinants are experienced as important by other healthcare professionals responsible for the implementation of healthcare improvements.

The use of determinant frameworks in practice is also restricted by the limited guidance available on how to analyse determinants and how to match determinants with implementation strategies [8, 12]. Most frameworks of implementation strategies (e.g. Expert Recommendations for Implementing Change (ERIC) [13]) have not linked their implementation strategies to determinants. This can lead to a mismatch between identified determinants and strategies [14], making the effect of implementation strategies variable [8]. A systematic review of 32 studies [15] showed that strategies tailored to determinants were more effective than those that were not; however, the methods used to select implementation strategies were often not well described [15]. Enhancing the link between identified determinants and implementation strategies is therefore a priority for implementation science [16].

To our knowledge, there is no relatively short general determinant tool with prioritised determinants based on the experienced importance according to healthcare professionals who are responsible for QI initiatives in their own practice. Such a tool could support these professionals to have a structured discussion regarding which determinants are most important to consider during different moments in the implementation process of the QI initiative and to find ways to analyse and address these determinants. The aim of this study was therefore to develop a tool of prioritised determinants based on the experienced importance according to healthcare professionals leading QI initiatives in their own workplace. In this way, this tool helps to bridge the research to practice gap by taking a serious look at how practical research-centric determinant frameworks are for those engaged in QI initiatives in the field. The tool was developed to be practical in the field rather than comprehensive, resulting in a relatively short list of determinants with suggestions for how to analyse and address them. In this article, we describe the development of this tool.

## Methods

### Setting

**Fig. 1:**
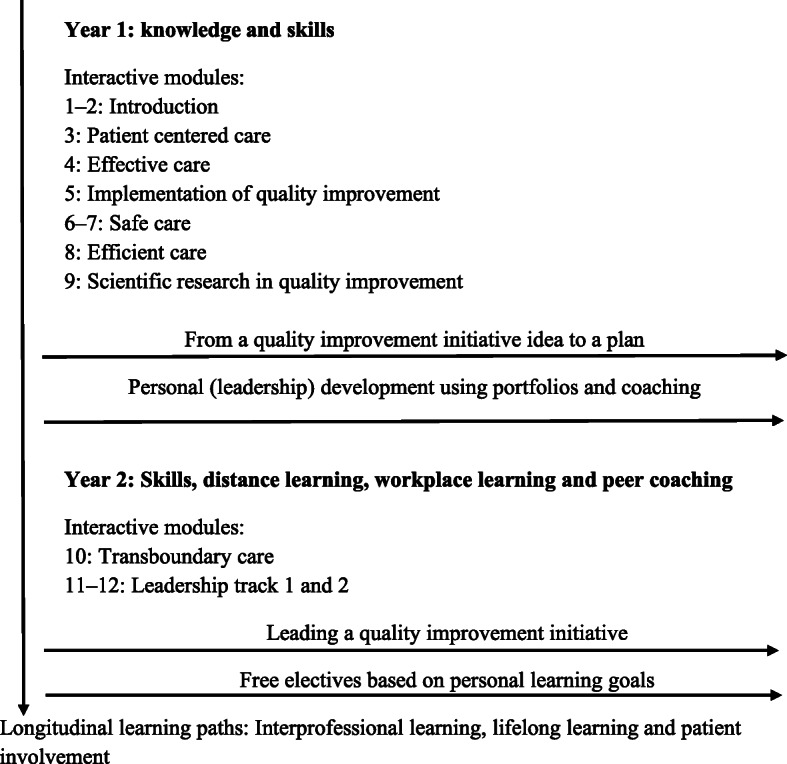
Elements of the Dutch two-year post-initial master’s on Quality and Safety in Healthcare

Healthcare professionals and implementation experts participated in this study. Both groups were involved in a 2-year half-time post-initial scientific master’s programme (subsequent to an initial master’s) on QI in healthcare. The programme trains healthcare professionals with an academic background to become leaders in the evidence-based improvement of healthcare quality and safety. Professionals are enrolled in 12 learning modules (see Fig. 1, which shows the core elements of the programme). The implementation experts are teachers and supervisors in these modules and have different specialties, such as leadership or patient involvement in QI. Professionals work intensively on their personal (leadership) development in QI through portfolios and coaching. Together, these educational interventions support professionals to perform a QI initiative at their workplace during their master’s programme. Because the initiatives are carried out in the context of a teaching programme, they are to some extent standardised (e.g. all professionals receive methodological support, all initiatives are performed in a hospital setting and are led by healthcare professionals, and all theses are evaluated using the Standards for Quality Improvement Reporting Excellence (SQUIRE) guidelines [17]), making them valuable sources for the development of this tool.

### Design

We used a mixed-methods design [18] to develop the tool. First, we performed a cross-sectional study using an online survey among healthcare professionals from the master’s programme, in which they were asked about the determinants they experienced as most facilitating and hindering during the performance of their QI initiative. After including the determinants in our tool, we performed semi-structured interviews with implementation experts about how healthcare professionals can analyse and address the determinants identified in their initiative. We used the Strengthening of Reporting of Observational Studies in Epidemiology guidelines (STROBE) and the Consolidated Criteria for Reporting Qualitative Research guidelines (COREQ) when designing our study. For more details, see Additional files 1 and 2.

### Part 1: Survey

#### Development

Two cohorts of healthcare professionals following the master’s programme (*N* = 41) were asked to fill in an online survey in which they were retrospectively asked to express the five most facilitating and five most hindering determinants they experienced during the performance of their QI initiatives. See Additional file 3 for the complete survey (in Dutch).

To support healthcare professionals with which determinants to name in their top 5, we provided a pre-specified list with an overview of determinants that are described in the literature. This list was based on existing determinants models. After reviewing and considering the literature on determinants models, we based our pre-specified list on two models: MUSIQ [4] and the TICD model [2]. To the best of our knowledge, MUSIQ is the only model that includes determinants related to team-level QI, while the TICD model is based on a systematic review of other determinant frameworks (*including for example the Consolidated Framework For Implementation Research*), not including MUSIQ. We therefore assume that the combination of both models provides an overview of all unique determinants.

**Fig. 2:**
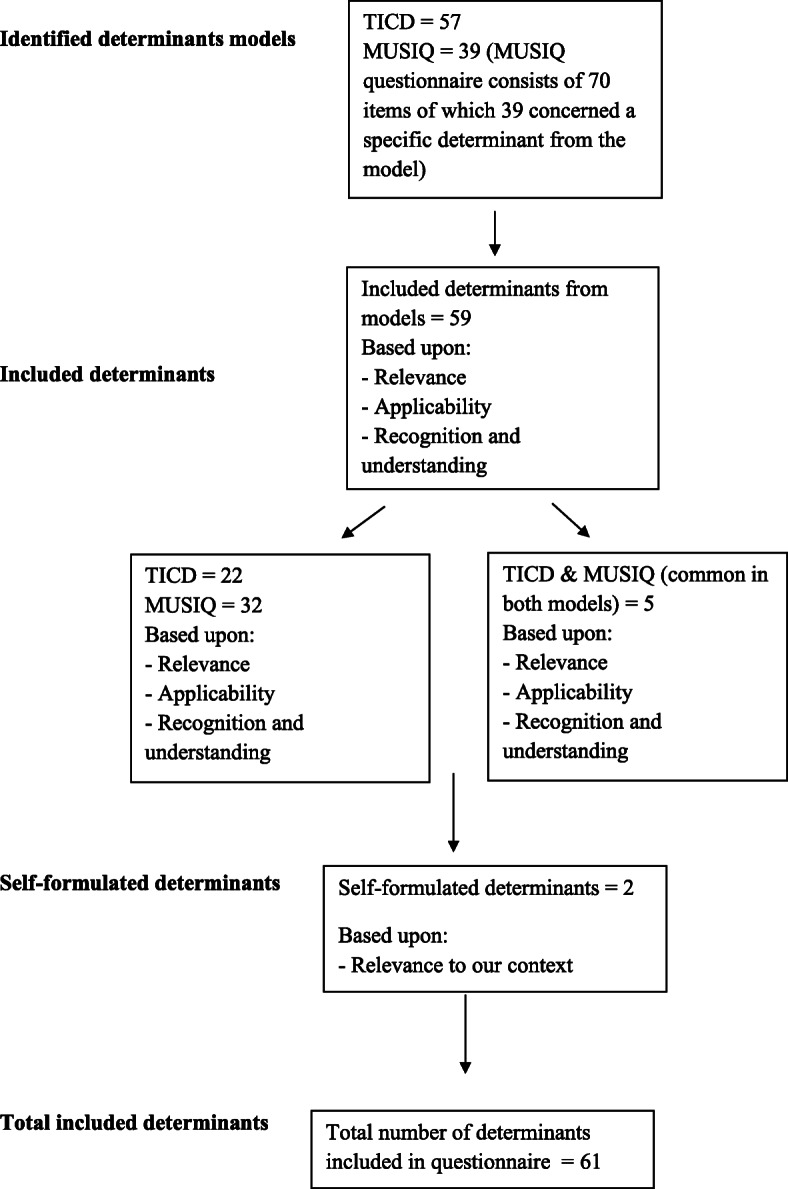
Flow diagram of the selection process of determinants in the pre-specified list

First, the researcher (AvT) combined the two models to gain insight into the unique and overlapping determinants. For the MUSIQ model, we used the questionnaire that was developed to measure contextual determinants [19]. For the TICD model, we used the TICD tool that was developed to facilitate the use of the model [2]. The researcher (AvT) and one member of the research team (HW) scored all determinants (57 TICD determinants, 39 MUSIQ determinants) by three criteria: relevance (the determinant should be of relevance for performing a QI initiative in healthcare), applicability (the determinant should be applicable across various relevant settings and types of improvement initiatives) and recognition (the determinant must be easily understood by healthcare professionals) [2]. Of the selected determinants, 32 came from the MUSIQ questionnaire, 22 from the TICD tool and five were identified in both tools. Two determinants were formulated by discussion with the research group based on their relevance to our context. We formulated all the included determinants as both facilitators and barriers, resulting in two lists of 61 determinants each. A flowchart of the selection process can be found in Fig. 2.

The translation and adaptation of the determinants for the pre-specified list was completed by researcher AvT. The list was extensively discussed with the entire research group. The determinants were organised according to the MUSIQ model levels: those of the external environment, organisation, microsystem and QI team. We also added two levels from the TICD model: the level of the patient and of the QI initiative itself.

#### Procedure

In 2014–2018, a total of 46 healthcare professionals with backgrounds in various medical and nursing specialties participated in the first two cohorts of the master’s programme. We included all healthcare professionals who started performing a QI initiative during their master’s. Professionals who dropped out of the programme and did not begin an initiative and those who explicitly stated that they did not want to participate were not recruited. In total, 41 healthcare professionals were recruited by e-mail to participate. Participation in this survey was voluntary. Informed consent was implied by the overt action of completing the online questionnaire after reading the information letter.

We administered the survey after the professionals, whether or not successfully, implemented their QI initiative. In the survey, professionals were retrospectively asked to name the five most facilitating and five most hindering determinants they experienced at any point during the performance of their QI initiative. Professionals could select determinants in two ways: choosing from the pre-specified list of facilitators and barriers and/or naming self-experienced determinants that were not included in this list. The survey could be completed in approximately 10 min.

#### Analyses

For the analyses, the participants’ self-experienced determinants were carefully evaluated by the researcher (AvT) to assess whether they could be merged with determinants from the pre-specified list or with determinants reported by other participants. Self-experienced determinants with the same meaning as a pre-specified determinant were accommodated within this determinant. Self-experienced determinants with the same meaning as another self-experienced determinant were combined, sometimes leading to a reformulation of the self-experienced determinant. The combined determinants were discussed with another member of the research team until consensus was reached (HW and/or HC).

A priority score was calculated for each of the top-five determinants named by the respondents. This score consisted of the sum of the ranking in the top five (i.e. a determinant ranked in first place scored 5 points, a determinant ranked fifth place scored 1 point), multiplied by the number of times a determinant was placed in a top five by professionals. Determinants with a priority score ≥ 20 were included in our tool.

### Part 2: Interviews

#### Questions

After prioritising each determinant and including the determinants with a priority score ≥ 20 in our tool, we performed semi-structured interviews with implementation experts. The purpose of these interviews was to gain insight on how to analyse and address the determinants in our tool, not further triangulation of which determinants are important. The interview guide consisted of open-ended questions about how healthcare professionals could be assisted to analyse determinants, use facilitators and confront barriers when performing a QI initiative. We also asked for general comments on the tool. The interview guide was pilot-tested with a member of the research team (HC) and adapted where necessary.

#### Procedure

We recruited 29 implementation experts from eight Dutch universities. They all had a teaching role in the master’s programme and possessed different expertise regarding implementation science. We used purposive sampling to select implementation experts with varied expertise in this field. All 29 implementation experts were approached by e-mail to participate and were provided with information about the study. Oral consent was recorded. The primary researcher (AvT) interviewed all implementation experts. All interviews were audio-taped and field notes were made.

During the interview, implementation experts were asked to choose two facilitators and two barriers from the tool, on which they wanted to provide input. Input was based on the implementation experts’ theoretical knowledge and practical experiences with QI. As an increasing number of interviews were held, it became clear that some determinants had received more input than others. Therefore, as we conducted more interviews, we asked implementation experts if they could also advise on the determinants which had previously received less input.

#### Analyses

All interviews were transcribed verbatim by the primary researcher (AvT), and from each transcript, an extensive summary was made to facilitate the analyses. The transcript summaries were analysed in pairs using principles from thematic analysis [20]. The primary researcher (AvT) analysed all summaries and two researchers (HW and HC) with implementation expertise also analysed half of the summaries each. This involved carefully reading the summaries before discussing the meaning of the input to identify and develop themes, which were reformulated into suggestions for analysing and addressing the determinants in the tool.

## Results

### Survey

Of the 41 professionals who received the survey, 28 responded (68% participation rate). Most participants had a professional background as a physician (50%), followed by a background as a nurse (36%). Tables 1 and 2 show characteristics of the respondents.

**Table 1:** Characteristics of healthcare professionals participating in the survey (*N* = 28)

Characteristics	***N*** (%)
**Professional background**	
Physician	14 (50%)
Nurse	10 (36%)
Pharmacist	2 (8%)
Healthcare scientist	1 (4%)
Healthcare jurist	1 (4%)
**Gender**	
Female	18 (64%)
Male	10 (36%)
**Hospital setting of their quality improvement initiative**	
Academic medical center	21 (75%)
Teaching hospital	6 (21%)
General hospital	1 (4%)
**Medical department of their quality improvement initiative**	
Surgery	6 (21%)
(Pediatric) intensive care	6 (21%)
Internal medicine	2 (7%)
Anesthesiology	1 (4%)
Emergency	1 (4%)
Geriatrics	1 (4%)
Gastrointestinal liver	1 (4%)
Neonatology	1 (4%)
Neurology	1 (4%)
Oncology	1 (4%)
Orthopedics	1 (4%)
Pediatrics	1 (4%)
Radiology	1 (4%)
Rehabilitation	1 (4%)
Rheumatology	1 (4%)
Transcending departments	1 (4%)
Urology	1 (4%)

**Table 2:** Improvement goals of healthcare professionals’ quality improvement initiatives (*N* = 28)

Improvement goal	
1. Reduction of medication administration errors 2. Improving early detection of deterioration amongst children 3. Improving resuscitation and non-technical team skills of nurses 4. Improving professional skills of nurses in using echography 5. Improving the use of the intra-hospital checklist by transporting patients 6. Improving the employability and well-being of healthcare professionals 7. Improving the use of the ‘Utrecht Symptoom Dagboek’ (Utrecht Symptoms Diary) 8. Improving hand hygiene compliance 9. Improving the amount of information patients remember during consultation 10. Improving the collection of patient experiences 11. Reduction of (extreme) pain of children below the age of 10 12. Improving shared decision making between parents and healthcare professionals 13. Improving patient transfer from intensive care 14. Reduction of the amount of unplanned readmissions 15. Reduction of acetylcysteine and/or salbutamol without a strict clinical indication 16. Improving shared decision making 17. Improving sleep quality on intensive care 18. Improving the medical handover in complex cases 19. Improving safety of preoperative agreements 20. Reduction of deep postoperative wound infections 21. Improving system awareness, communication skills, empathy and professional identity 22. Improving patient satisfaction by substitution of care to outreach clinics 23. Improving patient identification and verification procedure 24. Reduction of intravenous medication errors 25. Improving the performance of STOP moments during the perioperative process 26. Improving wound care after hand operations 27. Improving nurses as role model in evidence based practice 28. Reduction of complications in admitted older patients	

We found 130 unique determinants named by respondents, consisting of 64 facilitators (53%) and 66 barriers (47%). Facilitators related to the QI team were most commonly reported, while the most commonly reported barriers were related to the level of healthcare organisations. Of the 64 facilitators, 32 (50%) were self-reported by the professionals and thus outside our pre-specified list of determinants. Of the 66 barriers, 34 (52%) were self-reported. Additional file 4 provides all determinants that were ranked in the professionals’ top five lists.

Twelve facilitators were found to have a priority score ≥ 20 (min. 24, max. 288). Most facilitators were related to the level of the department. The availability of management support was identified as the most important facilitator. We found nine barriers with a priority score ≥ 20 (min. 20, max. 767). Most barriers were experienced at the organisation level. Healthcare professionals’ feeling they had insufficient time to perform the QI initiative was identified as the most important barrier. Table 3 provides a list of the 12 facilitators and nine barriers with their priority scores.

**Table 3:** Facilitators and barriers with a priority score ≥ 20, grouped by category

	Priority score^**a**^
**Facilitators**	
***External environment***	
Incentives or pressure (financial, legal or politica)	64
***Organisation***	
Sufficient support of expertise in the field of quality improvement	95
Culture of improvement	60
Sense of urgency^b^	42
Sufficient available time	90
***Department***	
Sufficient support of management	288
Employee support^b^	203
Bottom-up project approach^b^	96
Enthusiastic and supportive department head^b^	76
Workforce is motivated about the improvement initiative	33
***Quality improvement team***	
Sufficient participation in the decision-making process by team members	24
***Intervention of the initiative***	
Intervention fits in with current workflow	120
**Barriers**	
***Organisation***	
Insufficient available time	767
Data infrastructure	120
Insufficient support of the Executive Board for the initiative	110
Opponents of the initiative	30
Other organisational changes (reorganisation, merger)	27
Insufficient integration of quality improvement	27
***Intervention***	
Lack of evidence in literature of the effects of intervention	24
***Department***	
Insufficient motivation among the workforce	21
Experiencing one’s competencies needed for the intervention as insufficient	20

^a^The priority score consists of (1) the sum of the ranking in the top five (i.e. a determinant ranked first place in the top five got 5 points, while a determinant ranked fifth place in the top five received 1 point) multiplied by (2) the number of times a determinant was placed in the top five by professionals

^b^Self-experienced determinant listed by professionals (not included in the pre-specified list of determinants used in the survey)

### Interviews

We interviewed 16 implementation experts (response rate = 55%). Reasons for non-participation were needed to meet other deadlines and overlapping expertise. Five interviews were conducted face to face at the professional’s workplace, while the remainder were held over the telephone. The interviews took on average 34 min (range 20–57 min). Table 4 shows the areas of expertise of the implementation experts.

**Table 4:** Area of expertise implementation experts participating in interviews

Area of expertise	***N*** implementation experts^**a**^
Qualitative research	1
Governance of quality and safety in healthcare	2
Patient safety and teamwork	2
Quality and safety advisor	4
Quality improvement in patient care and education	1
Evidence based healthcare	2
Implementation improvement projects in patient care	2
Implementation as learning	2
Implementation in the field of perioperative patient safety	3
Evaluation of quality improvement	1
Patient involvement	3

^a^Some experts were interviewed from different areas of expertise

For each determinant with a priority score **≥** 20, we identified several suggestions on (1) how to assist healthcare professionals in analysing the determinant and (2) how to use a facilitator or tackle a barrier. The suggestions are included in the tool under the headings ‘analyse determinant’ and ‘address determinant’.

In addition to providing input on how to analyse and address the determinants in our tool, experts frequently made general comments about performing QI initiatives. These comments led to the identification of four themes: communication, keep it small, influence and concern, and learning by doing. Table 5 provides a description of each theme.

**Table 5:** Themes identified from interviews with implementation experts on how to analyse and address determinants for performing QI initiatives

**Communication:** It seems important to communicate about the initiative to different stakeholders at different levels both inside and outside the organisation. Depending on the stakeholder, this communication differs in terms of how much information the project leader provides, which information is provided and the way in which the information is provided. Experts talked about *‘speaking the language of the stakeholder*’. It is important to express what is expected from stakeholders, to periodically inform them about progress and to listen carefully to what they say about the initiative. Creating room for stakeholders to ask questions about the initiative is also considered important. A communication plan can facilitate the structuring of this communication. **Keep it small:** Experts emphasised the importance of keeping an initiative small, both in terms of the goal(s) and the participants and departments to be included. Project leaders should look at the initiative as one part of the whole QI process in their institution and try to improve something in their microsystem. Successful implementation in this context can lead to future spread and sustainability. **Influence and concern:** This theme relates to focusing on determinants that can be influenced by healthcare professionals as improvement leaders, rather than determinants that lie beyond their influence. Determinants that can only be addressed with disproportionate effort will result in a disbalance between effort and result, which can lead to an energy leak. **Learning by doing:** Experts frequently noticed that the implementation of an QI initiative is a process that is equally or even more important than the beneficial outcomes. One expert said: ‘*the journey itself is much more important than your end goal. Projects can fail in terms of outcomes, but you can learn so much of that by putting value on the process*’. By seeing the implementation process as a learning process, healthcare professionals can learn for future initiatives	

All facilitators and barriers with a priority score ≥20, together with suggestions for analysing and addressing the determinants, were combined in a practical tool. Based on the major themes that emerged from interviews with implementation experts, a distinction is made between determinants that lie within the control of healthcare professionals (impact) and determinants that lie beyond their control (involvement). Table 6 shows one elaborate facilitator on the department level and one elaborate barrier on the department level from the tool. The full tool can be found in Additional file 5.

**Table 6:** An elaborate barrier and facilitator from the tool

**Hindering determinants**	**Target population**	**Analysis (diagnosis)**	**Range of impact or involvement**^**1**^	**Approaches (how do I make sure the QI initiative is hindered as little as possible by these determinants)**
*Department level*				
Insufficient motivation among the workforce (21^2^)	Employees who have to work with the intervention	* Do employees show commitment to the QI initiative, e.g. by being present at meetings, honouring agreements, and having the QI initiative discussed during work meetings? * Are employees saying that they are not motivated to work on the QI initiative?	Impact	* Make the employee take ownership of the problem, e.g. by giving them practical examples or letting them experience the problem from their own perspective * Clearly show that the QI initiative has been integrated with other issues in the department by making it clear how the project fits in with other ongoing projects or things happening in the department * Open the personal interests and underlying reasons for employees’ lack of motivation up for discussion by approaching them individually. A facilitating approach is helpful: ask questions such as “what can I do for you in order to convince you to participate in this initiative”? * Proactively inform department management about the QI initiative and ask them to bring the initiative t to the attention, such as by sending the newsletter or addressing it at the start of the day
**Facilitating determinants**	**Target population**	**Analysis (diagnosis)**	**Range of impact or involvement**^**1**^	**Implementation (how do I make sure this determinant is present in my QI initiative)**
*Department level*				
Sufficient support of management (288^2^)	Department management	* Conduct a stakeholder analysis * Does management meet its agreements? Are you involved in matters relating to the initiative? Do you receive information about the initiative? * Are a member of the Executive Board and a department head the project sponsor? Do they actively support it and provide resources?	Impact	* Seek support from management before the start of the initiative * At least once every 9 months, according to an agreement with the management, provide periodic information about the progress of the initiative. This is part of a detailed communication plan * Explicitly ask the department head and a member of the Executive Board to sign the project plan

*QI* quality improvement

^1^The circle of impact contains the elements/people/contexts that you can have an influence on. The circle of involvement contains elements/people/contexts that you are involved by but that you do not have any influence on or where it is difficult to influence things

^2^The priority score of the determinant. This priority score consisted of the sum of the ranking in the top five (i.e. a determinant ranked in first place scored 5 points, a determinant ranked fifth place scored 1 point), multiplied by the number of times a determinant was placed in a top five by professionals. Determinants with a priority score ≥ 20 were included in our tool

## Discussion

In this study, we developed a tool of 12 prioritised facilitators and nine prioritised barriers based on the experienced importance according to healthcare professionals performing QI initiatives in practice. For every determinant in the tool, suggestions on how to analyse and address these determinants are provided. This tool can be used before, during and after the implementation of a QI initiative to guide discussion on which determinants are important to consider. The tool therefore helps healthcare professionals to learn from failures and successes, which can be used in future initiatives. Most facilitators in our tool are at the level of the department, while most barriers are at the organisational level. We identified support from the departmental management staff as the most important facilitator, while lacking the proper time to perform an initiative was the most important barrier. Methods for analysing and addressing each determinant are provided, based on interviews with implementation experts. Although not every determinant can be directly influenced by professionals, good communication with stakeholders, keeping the initiative small and learning from implementation are important overall recommendations for performing QI initiatives.

### Differences with other determinant models

In contrast to most determinant models in the literature, our tool makes an explicit distinction between barriers and facilitators. We assume that the presence of a determinant during implementation does not necessarily have to be equally as helpful as its absence would be prohibitive, and vice versa. By analysing the professionals’ top five most important experienced facilitators and barriers, we conclude that this assumption is valid because we found that determinants that are experienced as most important facilitators were different than determinants that are experienced as most important barriers. Our tool therefore contains separate lists of prioritised facilitators and barriers. We hope that this distinction supports professionals in making more informed choices on which facilitators to use and which barriers to address during the implementation of a QI initiative.

Because identifying determinants is the first step to challenge or use determinants, we not only include a list of prioritised facilitators and barriers but also provide practical suggestions on how to analyse determinants, use facilitators and address barriers. Other  determinant models often fail to provide a link between determinants and the strategies to address them [8, 15, 16]. A recent study of which implementation strategies in the ERIC model would best address contextual barriers from the CFIR found that respondents had varying opinions regarding which implementation strategies best addressed each contextual barrier [12]. This result can be explained by the fact that professionals chose implementation strategies without fully understanding the determinant and that most implementation strategies are limited in their specification, poorly described and ‘package’ approaches consisting of multiple poorly understood elements [21]. Our tool first helps to address these concerns by including suggestions how to analyse the determinants. By applying those analysing methods, there will be a better understanding of the determinant leading to more informed choices on selecting implementation strategies. Thereby, the practical suggestions in our tool to address the determinants are different to those of other implementation strategies in that they are concrete and focus on one specific determinant. Future research should strengthen our suggestions to address the determinants by enhancing the evidence for these suggestions. Also linking specific behavioural change strategies, based on theoretical constructs, to the determinants in our tool would be a valuable future step.

### Reflection on the barriers and facilitators in the tool

We identified receiving sufficient support from management as the most important facilitator. Support from management helps to formally confirm an initiative, for example by integrating it in the policy statements of the department [22] or by providing resources [19]. In an exploratory analysis of the MUSIQ model, researchers found that microsystem determinants (e.g. department-level factors) have direct effects on the success of QI initiatives [19]. Microsystem leadership (similar to our determinant ‘management support of department’) was not found to have a direct influence on success; however, this determinant was found to be directly influenced by QI team leadership, which in turn had one of the strongest direct effects on measures of success. Our tool is based on the experiences of QI team leaders, who, it could be argued, are highly influenced by their departmental management team.

Analysis of the MUSIQ model also found that most determinants related to the QI team had a direct effect on success because this team is responsible for guiding the implementation [19]. Our results also show that facilitators at the level of the QI team were most often reported in professionals’ top 5 but were not included in our tool due to their priority score (< 20). This result could mean that the various individual determinants related to the QI team are independently not experienced as most important but that these determinants together could make the total synergistic importance of this domain very high. Future research on this tool should study how and why the QI team is important in the performance of a QI initiative.

Experiencing insufficient time to perform the initiative was reported as the most important barrier by healthcare professionals, which is in line with previous study results. A study of barriers to healthcare providers’ adherence to guidelines, diffusion of innovation and implementation of evidence into practice found time to be the most common resources-related barrier [23]. Furthermore, a systematic review on the barriers to evidence-based medicine identified lack of time as one of the most common barriers [24]. However, based on our interviews with implementation experts, lack of time seems to be a ‘surface’ barrier, underlying multiple causes. Although the workload of healthcare professionals is a probable reason for this determinant being so highly prioritised [24], this feeling may also be grounded in other causes that may be the real experienced barrier As we also formulate in our tool, we suggest that professionals first analyse what causes the lack of time and then find an appropriate solution for this cause.

Our results showed that more than half of the determinants named in the top 5 were self-experienced (i.e. outside our pre-specified list of determinants). Furthermore, our tool includes four facilitators that were self-experienced by the professionals. These facilitators include urgency to improve, bottom-up approach, an enthusiastic and supportive head of department and workforce commitment. A bottom-up approach to implementation was one of the most highly prioritised facilitators. Some of these ‘new’ determinants are not new in other disciplines; for example, a bottom-up approach is a frequently mentioned theme in literature about policy implementation [25, 26].

It is worthwhile to discuss whether the self-experienced determinants we found are truly ‘new’. We can reflect on this result from a methodology point of view. During the analysis of the determinants from the top 5, the researchers decided whether the determinant belonged to a determinant from the pre-specified list (being not ‘new’) or that a determinant did not belong to a determinant from this list (being ‘new’). This decision was based on the interpretation, experiences and expertise of the researchers and therefore not fully objective. However, every merged determinant was discussed with a second researcher with implementation expertise.

Another explanation is that the determinants we identified as ‘new’ have the same meaning as determinants that already exist in frameworks but are phrased in different wordings. It could be that different languages are used between practitioners in the field and researchers when they talk about determinants. This is an important finding because when determinants are interpreted differently by research and practice, the terms used in existing frameworks may be meaningless or unrecognisable for those in practice, leading to less applicability of these frameworks and a bigger gap between research and practice. If this is the problem, we should try to address it explicitly so determinant frameworks can be used by those in practice.

Finally, it could be that existing determinant frameworks do not include all determinants that are experienced as important in practice. This could result from the fact that most existing frameworks are not developed with or based on experiences of people responsible for implementing QI initiatives in practice. Most frameworks are developed using expert meetings and literature reviews. This could lead to a narrow and one-sided view on determinants. If this is the case, we should try to address it by developing frameworks with the involvement of those performing QI in field, such as healthcare professionals. Based on our results, we cannot be sure whether the determinants we labelled as ‘new’ are truly ‘new’. Therefore, we suggest future research to perform a structured conceptual analysis on the determinants to decide whether they are truly new or concern new wordings for the same construct.

During the interviews, several experts highlighted the need to consider QI initiatives as a learning process in itself. Although in healthcare implementation science this view on QI is relatively new, in other fields such as human recources and behavioural science this view is already widely supported [27]. It is important to understand what kind of learning process takes place during the implementation of a QI initiative to support the initiative [28]. There are some important design principles that support these learning processes [27]. It is notable that some of the principles are reflected in the determinants in our tool; for instance, the principle ‘working from individual motivation’ is in line with our determinant relating to the motivation of the workforce. The design principle ‘creating something together’ is in line with our facilitator of a ‘bottom-up approach’ and helps to create a common practice. Finally, the principle ‘organising creative turmoil’ is similar to our facilitator ‘sense of urgency’ [27]. It is also important to note that these determinants that support learning from the implementation process are also reflected in adaptive leadership behaviours that seems crucial for QI initiatives to achieve their goals [29]. The similarities between the determinants we found and the design principles [27] and adaptive leadership behaviours [29] suggest to place more emphasis on considering implementation as a learning process and using adaptive leadership behaviours during implementation. Process evaluations of QI initiatives currently focus on how the initiative was delivered in a specific context to interpret and explain outcomes [30]. It would be a valuable addition to these evaluations to assess what healthcare professionals, and other stakeholders, have learnt from the implementation, especially from overcoming barriers.

### How to use this tool

By providing a tool of prioritised facilitators and barriers with practical suggestions for how to analyse and address determinants, we intend to help healthcare professionals to have structured discussions regarding which determinants are important to consider during different moments in the implementation process of the QI initiative. Using the tool prior to the implementation can help to identify potential barriers and facilitators to implementation, adapt the initiative before implementation and consider how learning can be supported. During implementation, our tool can serve to monitor the implementation and to identify and address determinants. After implementation, the tool can help to reflect upon which determinants influenced the implementation. Although we recommend to use our tool during different phases of the implementation, we did not take into account that the experienced importance of determinants will vary throughout the lifetime of a QI imitative in the development of the tool. We recommend future research to ask professionals at different moments during the implementation of the initiative which determinants they experience as most important.

Our determinants are prioritised based on the experiences of healthcare professionals who were leading a QI initiative in the hospital setting. It is assumed that the higher the priority score, the more important this determinant is in the implementation of the initiative. However, as every initiative is unique and every context is different, it is not definite that the determinants we found will also be reflected in other contexts or initiatives. It is therefore important that QI teams have a structured discussion about the determinants in our tool to discuss whether the determinants are reflected in their context. Although the science of measuring determinants is immature [1], the column ‘analysis’ in the tool will be helpful for this task. During those discussions professionals should also think about other possible determinants that are important in their context and add them to this tool. Additional file 4 which provides all determinants that were ranked in professionals’ top five could support these discussions.

Knowing which determinants could potentially influence the implementation does not mean that the determinant can be addressed by healthcare professionals; interviews with implementation experts showed that some determinants lie beyond the control of healthcare professionals. An organisational culture supportive of QI is an example of a facilitator, but is difficult to influence directly and in the short term by healthcare professionals. However, this does not imply that these determinants should be fully ignored by healthcare professionals. We recommend professionals to give priority on addressing the determinants that lie within their control yet at the same time do not lose sight on the determinants beyond their control [31]. First addressing the determinants within practitioners’ control will help to build motivation and commitment to the QI initiative. These ‘early wins’ could also help to address or use the determinants that are beyond their influence by stimulating the organisation to influence those determinants. Also, these determinants that lie beyond practitioners’ control should be kept on the horizon, knowing that they won’t change quickly but by the use of adaptive leadership behaviours and other strategies small steps in the right direction can be obtained. Because our tool is one of the first that includes the sphere of influence, we recommend more research on which determinants can be influenced by whom and under which circumstances.

### Limitations

Our study has several limitations. The first limitation has to do with the context dependency of our tool, limiting the generalisability. The QI initiatives at the basis of this study were performed within the context of an educational programme, making the results susceptible to selection bias. It is possible that healthcare professionals performing a QI initiative outside the context of an educational programme will experience different determinants. Although this possibility, our initiatives are performed using the principles from experiential learning which means that real-life experiences in the context relevant to learners own future career are used, making the difference with initiatives performed outside the educational context relatively small [32, 33]. Thereby, all of the included improvement initiatives are performed within the Dutch hospital setting. We propose future research to test this tool in other contexts, to see whether the determinants we found are also experienced as most important in those contexts. Also, our tool is based on a small number of QI initiatives (*N* = 28), which may further limit its generalisability. The initiatives were performed in a wide range of hospitals across the Netherlands however, including all academic hospitals in the country. Another limitation is that there could be a potential bias in the participants' top 5 barriers and facilitators. Respondents could have identified the easier well-known determinants more easy and overlooked the less known determinants that could potentially have a greater impact. However, we tried to minimalise this bias by letting participants choose determinants from our pre-specified list of determinants which included also the less obvious determinants. Finally, our tool has not been prospectively validated. Further evaluation and modification is needed, including feedback from a broad range of healthcare professionals about their experiences with the tool. This tool is not a finished product and will benefit from further adjustments and developments.

## Conclusion

To our knowledge, this is the first tool of prioritised determinants based on the experiences of healthcare professionals in practice. Our tool is not a substitute for the comprehensive well-developed frameworks that exists but is complementary to these frameworks in that it helps bridging the research to practice gap by including those determinants that are experienced as most importance during the performance of a QI initiative by those engaged in QI in the field. The tool consists of nine barriers and 12 facilitators for the performance of QI initiatives. For each of these barriers and facilitators, suggestions on how to analyse and address the determinant are given based on input from implementation experts. This tool will facilitate healthcare professionals in the systematic reflection on determinants for QI initiatives; however, additional research is needed to further develop and validate our tool.

## Supplementary information

**Additional file 1.** STROBE checklist.

**Additional file 2.** COREQ checklist.

**Additional file 3.** Determinant survey (Dutch).

**Additional file 4.** Tables with facilitators and barriers.

**Additional file 5.** Tool determinants QI initiatives.

## Data Availability

The datasets that support the findings of this study are not publicly available due to information that could compromise research participant consent and privacy but can be made available from the corresponding author (AvT) with appropriate precautions and upon reasonable request.

## Abbreviations

QI: Quality improvement
TICD: Tailored Implementation for Chronic Disease
CFIR: Consolidated Framework for Implementation Research
MUSIQ: Model for Understanding Success in Quality
MIDI: Measurement Instrument for Determinants of Innovations
ERIC: Expert Recommendations for Implementing Change
SQUIRE: Standards for Quality Improvement Reporting Excellence
