# Supplementary material for: Development of a tool for identifying and addressing prioritised determinants of quality improvement initiatives led by healthcare professionals: a mixed-methods study
Source: Implement Sci Commun. 2020 Oct 23;1:92. doi: 10.1186/s43058-020-00082-w (PMC7584081; doi:10.1186/s43058-020-00082-w)
Supplement: Supplementary file 2 — Additional file 2. COREQ checklist. [file 43058_2020_82_MOESM2_ESM.docx]

**Additional file 2. COREQ 32-item checklist for manuscript “****Development of a tool for identifying and addressing prioritised determinants of quality improvement projects led by healthcare professionals: a mixed-methods study”**

| **No. Item** | **Guide questions/description** | **Reported on Page #** |
| --- | --- | --- |
| **Domain 1: Research team and reﬂexivity** |  |  |
| *Personal Characteristics* |  |  |
| 1. Interviewer/facilitator | Which author/s conducted the interview or focus group? | Methods/Interviews/Procedure  Page 14 |
| 2. Credentials | What were the researcher’s credentials? E.g. PhD, MD | Title page  Page 1 |
| 3. Occupation | What was their occupation at the time of the study? | Title page (department)  Page 1 |
| 4. Gender | Was the researcher male or female? | NA^1^ |
| 5. Experience and training | What experience or training did the researcher have? | Methods/Interviews/Procedure  Page 14 |
| *Relationship with participants* |  |  |
| 6. Relationship established | Was a relationship established prior to study commencement? | NA^1^ |
| 7. Participant knowledge of the interviewer | What did the participants know about the researcher? e.g. personal goals, reasons for doing the research | Methods/interviews/procedure  Page 14 |
| 8. Interviewer characteristics | What characteristics were reported about the inter viewer/facilitator? e.g. Bias, assumptions, reasons and interests in the research topic | NA^1^ |
| **Domain 2: study design** |  |  |
| *Theoretical framework* |  |  |
| 9. Methodological orientation and Theory | What methodological orientation was stated to underpin the study? e.g. grounded theory, discourse analysis, ethnography, phenomenology, content analysis | Methods/interviews/analysis  Page 15 |
| *Participant selection* |  |  |
| 10. Sampling | How were participants selected? e.g. purposive, convenience, consecutive, snowball | Methods/interviews/procedure  Page 14 |
| 11. Method of approach | How were participants approached? e.g. face-to-face, telephone, mail, email | Methods/interviews/procedure  Page 14 |
| 12. Sample size | How many participants were in the study? | Methods/interviews/procedure  Results/interviews  Page 17 |
| 13. Non-participation | How many people refused to participate or dropped out? Reasons? | Results/interviews  Page 17 |
| *Setting* |  |  |
| 14. Setting of data collection | Where was the data collected? e.g. home, clinic, workplace | Methods/interviews/procedure  Page 14 |
| 15. Presence of non-participants | Was anyone else present besides the participants and researchers? | NA^1^ |
| 16. Description of sample | What are the important characteristics of the sample? e.g. demographic data, date | Methods/interviews/procedure  Page 14  Results/interviews  Page 17 |
| *Data collection* |  |  |
| 17. Interview guide | Were questions, prompts, guides provided by the authors? Was it pilot tested? | Methods/interviews/questions  Page 13/14 |
| 18. Repeat interviews | Were repeat interviews carried out? If yes, how many? | NA^1^ |
| 19. Audio/visual recording | Did the research use audio or visual recording to collect the data? | Methods/interviews/procedure  Page 14 |
| 20. Field notes | Were ﬁeld notes made during and/or after the inter view or focus group? | Methods/interviews/procedure  Page 14 |
| 21. Duration | What was the duration of the interviews or focus group? | Results/Interviews  Page 17 |
| 22. Data saturation | Was data saturation discussed? | Methods/interviews/ procedure  Page 14 |
| 23. Transcripts returned | Were transcripts returned to participants for comment and/or correction? | NA^1^ |
| **Domain 3: analysis and ﬁndings** |  |  |
| *Data analysis* |  |  |
| 24. Number of data coders | How many data coders coded the data? | Methods/interviews/analysis  Page 15 |
| 25. Description of the coding tree | Did authors provide a description of the coding tree? | Methods/interviews/analyses/  Page 15 Results/interviews  Page 17, 18 and 19 |
| 26. Derivation of themes | Were themes identiﬁed in advance or derived from the data? | Methods/interviews/analyses  Page 15 Results/interviews  Page 17, 18 and 19 |
| 27. Software | What software, if applicable, was used to manage the data? | NA^1^ |
| 28. Participant checking | Did participants provide feedback on the ﬁndings? | NA^1^ |
| *Reporting* |  |  |
| 29. Quotations presented | Were participant quotations presented to illustrate the themes/ﬁndings? Was each quotation identiﬁed? e.g. participant number | Results/interviews  Page 17, 18 and 19 (We rarely used quotations because the themes that derived from the data were reformulated in concrete suggestions to analyze and address determinants in our tool) |
| 30. Data and ﬁndings consistent | Was there consistency between the data presented and the ﬁndings? | Results/ interviews  Page 17, 18 and 19 |
| 31. Clarity of major themes | Were major themes clearly presented in the ﬁndings? | Results/interviews  Page 19 |
| 32. Clarity of minor themes | Is there a description of diverse cases or discussion of minor themes? | Results/interviews  Page 17, 18 and 19 |

^1^ NA indicates not applicable
