# Supplementary material for: Development of a tool for identifying and addressing prioritised determinants of quality improvement initiatives led by healthcare professionals: a mixed-methods study
Source: Implement Sci Commun. 2020 Oct 23;1:92. doi: 10.1186/s43058-020-00082-w (PMC7584081; doi:10.1186/s43058-020-00082-w)
Supplement: Supplementary file 3 — Additional file 3. Determinant survey (Dutch). [file 43058_2020_82_MOESM3_ESM.docx]

**Additional file 3. Determinant survey (Dutch)**

Vragenlijst evaluatie bevorderende en belemmerende factoren uitvoering kwaliteitsverbeterproject NFU-master ‘Kwaliteit en Veiligheid in de Patiëntenzorg’

*Meest belangrijke bevorderende factoren*

Geef een top vijf van factoren die u het meest geholpen hebben in de uitvoering van uw project. Hierbij is nummer 1 de meest bevorderende factor. Wanneer u geen vijf factoren kunt noemen, zijn minder factoren ook voldoende. Als hulpmiddel vindt u op de volgende pagina een lijst met mogelijke bevorderende factoren afkomstig uit een drietal modellen over determinanten voor kwaliteitsverbetering: het Tailord Implementation for Chronic Diseases model (Flottorp et al., 2013), het Model for Understanding Succes in Quality (Kaplan, 2011) en het Meetinstrument voor Determinanten van Innovaties (Fleuren et al., 2012). Deze lijst is niet compleet dus het is mogelijk dat u andere, niet in de lijst genoemde factoren, heeft ervaren.

1. …..
2. …..
3. …..
4. …..
5. …..

**Afdeling**

Let op: het gaat hier niet om de leden uit het projectteam maar om de medewerkers van de afdeling die de interventie uit uw project hebben moeten toepassen.

- Voldoende ondersteuning door management uitvoering KVP
- Positieve cultuur tegenover kwaliteitsverbetering
- Voldoende motivatie voor kwaliteitsverbetering medewerkers
- Voldoende expertise medewerkers met interventie
- Medewerkers bewust en bekend met interventie
- Medewerkers voldoende kennis over de eigen praktijk
- Voldoende instemming medewerkers met interventie
- Voldoende geloof medewerkers in verwachte uitkomsten interventie
- Ervaren eigen competenties medewerkers
- Medewerkers mogelijkheid tot plannen van veranderingen door KVP
- Terugkoppeling aan medewerkers over voortgang interventie
- Interventie persoonlijk voordeel voor medewerkers

**Kwaliteitsverbeter team**

- Aanwezigheid ‘echt’ team
- Uw eigen professionele achtergrond
- Voldoende diversiteit teamleden
- Aanwezigheid kwaliteitsverbeter expert in team
- Teamleden kenden elkaar vóór KVP
- Teamleden eerdere ervaring uitvoeren KVP
- Uw beschikbaarheid voor teamleden voldoende
- Voldoende deelname teamleden in besluitvorming
- Voldoende waardering individuele verschillen teamleden
- Verschillende ideeën voldoende overwogen
- Voldoende bijdrage teamleden aan KVP
- Overeenstemming teamleden over doel KVP
- Doel KVP leidend voor handelen teamleden
- Teamleden gedroegen zich zoals van hen verwacht werd
- Teamleden gecommitteerd aan dezelfde doelen KVP
- Voldoende kwaliteitsverbeter vaardigheden teamleden
- Voldoende geloof teamleden in verbetering door interventie KVP

**Patiënten**

- Voldoende behoefte aan KVP
- Voldoende kennis en vaardigheden
- Passende normen en waarden
- Voldoende motivatie deelname interventie KVP

**Kwaliteitsverbeterproject**

- Aanwezigheid bewijs verwachte effect(en) interventie
- Duidelijke omschrijving interventie
- Beschikbaarheid protocol/richtlijn/aanbeveling voor gebruiker
- Protocol/richtlijn/aanbeveling consistent met bestaande protocollen/richtlijnen
- Interventie passend binnen huidige workflow
- Effecten interventie voldoende zichtbaar voor gebruikersgroep

**Organisatie**

- Academie of periferie
- Voldoende ondersteuning van expertise op gebied van kwaliteitsverbetering
- Voldoende betrokkenheid RvB kwaliteitsverbeter activiteiten
- Voldoende ondersteuning KVP door lid RvB
- Positieve organisatie cultuur tegenover kwaliteitsverbetering
- Voldoende integratie kwaliteitsverbetering in organisatie
- Data infrastructuur
- Voldoende beschikbare financiële middelen
- Voldoende beschikbare tijd
- Voldoende ruimte voor training en onderwijs over kwaliteitsverbetering
- Voldoende waardering en beloning voor kwaliteitsverbetering
- Taakverdeling werknemers
- Beleid en regelgeving opgeschreven
- KVP passend bij strategische doelen
- Tegenstanders KVP
- Andere veranderingen organisatie (reorganisatie, fusie etc.)

**Externe omgeving**

- Beschikbare gezondheidszorg budget
- Bestaande regel- en wetgeving
- Financier beleid
- Invloedrijke personen
- Sponsoring (personeel, expertise, geld of andere middelen)
- Druk of prikkels

*Meest belangrijke belemmerende factoren*

Geef een top vijf van factoren die u het meest belemmerd hebben in de uitvoering van uw project. Hierbij is nummer 1 de meest belemmerende factor. Wanneer u geen vijf factoren kunt noemen, zijn minder factoren ook voldoende. Als hulpmiddel vindt u op de volgende pagina een lijst met mogelijke belemmerende factoren afkomstig uit een drietal modellen over determinanten voor kwaliteitsverbetering: het Tailord Implementation for Chronic Diseases model (Flottorp et al., 2013), het Model for Understanding Succes in Quality (Kaplan, 2011) en het Meetinstrument voor Determinanten van Innovaties (Fleuren et al., 2012). Deze lijst is niet compleet dus het is mogelijk dat u andere, niet in de lijst genoemde factoren, heeft ervaren.

1. …..
2. …..
3. …..
4. …..
5. …..

**Afdeling**

Let op: het gaat hier niet om de leden uit het projectteam maar om de medewerkers van de afdeling die de interventie uit uw project hebben moeten toepassen.

- Onvoldoende ondersteuning management KVP
- Negatieve cultuur tegenover kwaliteitsverbetering
- Onvoldoende motivatie voor kwaliteitsverbetering medewerkers
- Onvoldoende expertise medewerkers met interventie
- Medewerkers onvoldoende bewust en bekend met interventie
- Medewerkers onvoldoende kennis over de eigen praktijk
- Onvoldoende instemming medewerkers met interventie
- Onvoldoende geloof medewerkers in verwachte uitkomsten interventie
- Ervaren eigen competenties medewerkers
- Medewerkers onvoldoende mogelijkheid tot plannen van veranderingen door KVP
- Onvoldoende terugkoppeling over voortgang interventie
- Interventie onvoldoende persoonlijk voordeel voor medewerkers

**Kwaliteitsverbeter team**

- Afwezigheid ‘echt’ team
- Uw eigen professionele achtergrond
- Onvoldoende diversiteit teamleden
- Ontbreken kwaliteitsverbeter expert in team
- Teamleden kenden elkaar vóór KVP
- Teamleden onvoldoende eerdere ervaring uitvoeren KVP
- Uw beschikbaarheid voor teamleden onvoldoende
- Onvoldoende deelname teamleden in besluitvorming
- Onvoldoende waardering individuele verschillen teamleden
- Verschillende ideeën onvoldoende overwogen
- Onvoldoende bijdrage teamleden aan KVP
- Onvoldoende overeenstemming teamleden over doel KVP
- Doel KVP onvoldoende leidend voor handelen teamleden
- Teamleden gedroegen zich niet zoals van hen verwacht
- Teamleden onvoldoende gecommitteerd aan dezelfde doelen KVP
- Onvoldoende kwaliteitsverbeter vaardigheden teamleden
- Onvoldoende geloof teamleden in verbetering door interventie in KVP

**Patiënten**

- Onvoldoende behoeften aan KVP
- Onvoldoende kennis en vaardigheden
- Niet passende normen en waarden
- Onvoldoende motivatie deelname interventie KVP

**Interventie in kwaliteitsverbeterproject**

- Afwezigheid bewijs verwachten effect(en) interventie
- Onduidelijke omschrijving interventie
- Onvoldoende beschikbaarheid van protocol/richtlijn/aanbeveling voor gebruikersgroep interventie
- Protocol/richtlijn/aanbeveling onvoldoende consistent met bestaande protocollen/richtlijnen
- Interventie onvoldoende passend binnen huidige workflow
- Effecten interventie onvoldoende zichtbaar voor gebruikersgroep

**Organisatie**

- Academie of periferie
- Onvoldoende ondersteuning van expertise op gebied van kwaliteitsverbetering
- Onvoldoende betrokkenheid RvB kwaliteitsverbeter activiteiten
- Onvoldoende ondersteuning KVP door lid RvB
- Negatieve organisatie cultuur tegenover kwaliteitsverbetering
- Onvoldoende integratie kwaliteitsverbetering in organisatie
- Data infrastructuur
- Onvoldoende beschikbare financiële middelen
- Onvoldoende beschikbare tijd
- Onvoldoende ruimte voor training en onderwijs over kwaliteitsverbetering
- Onvoldoende waardering en beloning voor kwaliteitsverbetering
- Taakverdeling werknemers
- Beleid en regelgeving onvoldoende opgeschreven
- KVP onvoldoende passend bij strategische doelen
- Tegenstanders KVP
- Andere veranderingen organisatie (reorganisatie, fusie etc.)

**Externe omgeving**

- Beschikbare gezondheidszorg budget
- Bestaande regel- en wetgeving
- Financier beleid
- Invloedrijke personen
- Sponsoring (personeel, expertise, geld of andere middelen)
- Druk of prikkels
