# Supplementary material for: Development of a tool for identifying and addressing prioritised determinants of quality improvement initiatives led by healthcare professionals: a mixed-methods study
Source: Implement Sci Commun. 2020 Oct 23;1:92. doi: 10.1186/s43058-020-00082-w (PMC7584081; doi:10.1186/s43058-020-00082-w)
Supplement: Supplementary file 4 — Additional file 4. Tables with facilitators and barriers. [file 43058_2020_82_MOESM4_ESM.docx]

**Additional file 4. Tables with facilitators and barriers named in top 5 (N=28)**

| **Facilitators** |  | **Ranked in facilitators top-5 N^b^** | **Place in ranking^c^** | | | | | |
| --- | --- | --- | --- | --- | --- | --- | --- | --- |
|  |  |  | 1st | 2nd | 3rd | 4rt | 5th |  |
| **Department (N=12)** |  |  |  |  |  |  |  | |
| Sufficient support of management |  | 9 | 4 | 3 | 1 | 1 |  | |
| Sufficient pre-existing expertise with targeted condition |  | 2 |  | 1 | 1 |  |  | |
| Workforce is motivated about the improvement project |  | 3 |  | 2 | 1 |  |  | |
| Workforce familiar with intervention QIP |  | 1 |  | 1 |  |  |  | |
| Workforce believe intervention will lead to desired outcomes |  | 2 | 1 |  |  | 1 |  | |
| Incentives to adhere to intervention for workforce |  | 1 |  |  | 1 |  |  | |
| Employee support^a^ |  | 7 | 4 | 1 | 1 | 1 |  | |
| Availability of role models^a^ |  | 2 |  | 1 | 1 |  |  | |
| Strong management structure^a^ |  | 1 |  |  | 1 |  |  | |
| Bottum-up project approach^a^ |  | 6 | 1 | 1 | 1 | 1 | 2 | |
| Enthusiastic and supportive department head^a^ |  | 4 | 3 | 1 |  |  |  | |
| **Quality improvement team (N=22)** |  |  |  |  |  |  |  | |
| A ‘real team’ |  | 2 | 1 | 1 |  |  |  | |
| My (project leader) professional background |  | 2 |  |  |  | 1 | 1 | |
| Diversity in background and experiences |  | 2 | 1 |  | 1 |  |  | |
| Authority on quality improvement available in team |  | 1 |  | 1 |  |  |  | |
| Sufficient participation in decision-making process by team members |  | 3 |  |  | 2 | 1 |  | |
| Individual differences appreciated |  | 1 |  |  |  | 1 |  | |
| Commitment to the same project goals |  | 1 |  |  |  |  | 1 | |
| Believe that intervention will result in improvement |  | 2 | 1 |  | 1 |  |  | |
| Familiar with each other before QIP |  | 1 |  |  |  |  | 1 | |
| My (project leader) ongoing presence for the team |  | 2 |  |  | 2 |  |  | |
| Project leader is enthusiastic and takes care of support and guidance^a^ |  | 1 |  |  |  | 1 |  | |
| Early adaptor in team^a^ |  | 1 |  |  |  | 1 |  | |
| Team meetings with clear agenda^a^ |  | 1 |  |  |  | 1 |  | |
| My (project leader) own expertise^a^ |  | 1 |  | 1 |  |  |  | |
| My(project leader) own motivation for performing QIP^a^ |  | 2 |  |  | 1 |  | 1 | |
| My (project lader) own earlier experiences performing QIPs^a^ |  | 1 |  | 1 |  |  |  | |
| Involvement of patient in team^a^ |  | 1 |  |  |  | 1 |  | |
| Already existing team and meeting structure^a^ |  | 1 | 1 |  |  |  |  | |
| Passionate and competent team^a^ |  | 1 | 1 |  |  |  |  | |
| Feedback from and to team members^a^ |  | 2 |  |  | 2 |  |  | |
| **Patient (N=1)** |  |  |  |  |  |  |  | |
| Input and support of patients ^a^ |  | 1 |  |  | 1 |  |  | |
| **Intervention (N=9)** |  |  |  |  |  |  |  | |
| Good accessibility of protocol/guideline (intervention) |  | 1 |  |  | 1 |  |  | |
| Intervention fits in with current workflow |  | 6 | 1 | 2 | 1 | 2 |  | |
| Evidence available that supports expected outcomes |  | 2 | 1 |  | 1 |  |  | |
| Clear description of intervention |  | 2 |  |  |  |  | 2 | |
| Connected to earlier interventions^a^ |  | 1 |  |  |  |  | 1 | |
| Intervention multidisciplinary developed^a^ |  | 1 |  |  | 1 |  |  | |
| Direct feedback on results^a^ |  | 2 |  |  | 1 | 1 |  | |
| Intervention published in high state medical journal^a^ |  | 1 |  | 1 |  |  |  | |
| Goal of intervention was clear^a^ |  | 1 |  |  |  | 1 |  | |
| Content of QIP^a^ |  | 1 |  |  |  | 1 |  | |
| **Organizstion (N=13)** |  |  |  |  |  |  |  | |
| Academic or peripheral medical center |  | 1 |  | 1 |  |  |  | |
| Sufficient support of expertise in the field of quality improvement |  | 5 | 2 | 1 | 1 | 1 |  | |
| QIP aligned with key strategic goals |  | 2 |  | 1 |  |  | 1 | |
| Financial resources |  | 2 |  |  | 2 |  |  | |
| Sufficient time available |  | 6 |  | 2 | 1 | 1 | 2 | |
| Other changes in organization |  | 1 |  |  | 1 |  |  | |
| Culture of improvement |  | 4 | 2 |  | 1 | 1 |  | |
| Room for training and education in quality improvement |  | 1 |  |  |  |  | 1 | |
| Flat organization structure^a^ |  | 1 |  |  | 1 |  |  | |
| Structured knowledge transfer^a^ |  | 1 |  |  | 1 |  |  | |
| Sense of urgency^a^ |  | 3 | 2 | 1 |  |  |  | |
| Support of board of executives for Master program^a^ |  | 1 |  | 1 |  |  |  | |
| **External environment (N=7)** |  |  |  |  |  |  |  | |
| Sponsoring |  | 1 |  |  | 1 |  |  | |
| Pressures or incentives |  | 4 | 2 | 1 |  | 1 |  | |
| Legislation |  | 1 |  | 1 |  |  |  | |
| Healthcare budget |  | 1 |  | 1 |  |  |  | |
| Support form others’^a^ |  | 1 |  | 1 |  |  |  | |
| Peers^a^ |  | 2 |  |  |  | 2 |  | |
| Support of teacher from Master^a^ |  | 1 |  |  |  | 1 |  | |
| Relevance of stakeholders^a^ |  | 1 |  |  |  |  | 1 | |
| Stakeholders easily accessible^a^ |  | 1 | 1 |  |  |  |  | |
| Support from home^a^ |  | 1 |  |  |  |  | 1 | |

^a^ Self-experienced facilitators by professionals (not included in this way in our pre-specified list of facilitators used in the survey)

^b^ Number of times a facilitator was ranked in a top 5

^c^ Number of times a facilitator was ranked on a specific place in the top 5

| **Barriers (N=66)** | **Ranked in barriers top-5 N^b^** | **Place in ranking (N)^c^** | | | | |
| --- | --- | --- | --- | --- | --- | --- |
|  |  | 1st | 2nd | 3rd | 4th | 5th |
| **Department (N=14)** |  |  |  |  |  |  |
| Negative culture for quality improvement | 1 |  |  |  |  | 1 |
| Insufficient motivation among the workforce | 3 | 1 |  |  |  | 2 |
| Insufficient knowledge workforce about own practice | 1 |  | 1 |  |  |  |
| Insufficient believe that project will lead to desired outcomes | 1 |  |  | 1 |  |  |
| Experiencing once competencies needed for the intervention as insufficient | 2 | 2 |  |  |  |  |
| No incentives to adhere to intervention for workforce | 1 |  |  |  | 1 |  |
| Change in deparments’vision by change in management^a^ | 1 | 1 |  |  |  |  |
| Work schedule^a^ | 1 |  |  |  | 1 |  |
| QIP performed in my own social environment^a^ | 1 |  | 1 |  |  |  |
| No sense of urgency workforce^a^ | 1 | 1 |  |  |  |  |
| Inclusion problems^a^ | 1 |  |  |  | 1 |  |
| No support of supervisor/head of department^a^ | 1 |  |  | 1 |  |  |
| Change of workforce during implementation^a^ | 1 |  | 1 |  |  |  |
| The workforce^a^ | 1 | 1 |  |  |  |  |
| Insufficient time workforce^a^ | 1 | 1 |  |  |  |  |
| **Quality improvement team (N=13)** |  |  |  |  |  |  |
| My own availability for improvement team insufficient | 1 | 1 |  |  |  |  |
| Insufficient believe that project results in improvement | 2 |  |  |  | 2 |  |
| My own professional background | 1 |  | 1 |  |  |  |
| Team members did not work on QIPs before | 2 |  |  | 1 | 1 |  |
| Team member did not behave as expected to be | 1 |  |  | 1 |  |  |
| Team members were not committed to the same QIPs’ goals | 1 |  |  | 1 |  |  |
| Dedicated team members leaving the organization^a^ | 1 |  |  | 1 |  |  |
| Not enough people for measurements^a^  Loss of professional support^a^ | 1 |  |  |  | 1 |  |
|  | 1 |  | 1 |  |  |  |
| Not enough knowledge team members^a^ | 1 |  |  | |  | 1 |
| Team leader not working on department where QIP is performed^a^ | 1 |  |  |  | 1 |  |
| Team members find it difficult to hold colleagues account for adherence to intervention^a^ | 1 |  | 1 |  |  |  |
|  |  |  |  |  |  |  |
| **Patient (N=6)** |  |  |  |  |  |  |
| No need for QIP by patients  Effect measurement hampered by heterogenous patient population^a^ | 1  1 | 1 | 1 |  |  |  |
| How many information should we give patients?^a^ | 1 |  | 1 |  |  |  |
| Difficult to involve patient in project team^a^ | 2 | 1 |  | 1 |  |  |
| No experience with patient participation in new intervention^a^ | 1 | 1 |  |  |  |  |
| No central direction concerning patient participation^a^ | 1 |  |  | 1 |  |  |
| **Intervention (N=4)** |  |  |  |  |  |  |
| Lack of evidence in literature of the effects of intervention | 3 |  |  | 2 | 1 |  |
| Benefits intervention not observable for target group | 2 | 1 |  |  | 1 |  |
| Not clearly (e.g. lack sufficient detail) described | 1 |  |  |  |  | 1 |
| Intervention is seen as threat^a^ | 1 | 1 |  |  |  |  |
| **Organisation (N=22)** |  |  |  | |  |  |
| Academic or peripheral medical center | 1 |  |  | | 1 |  |
| Insufficient involvement in quality improvement activities board of directors | 1 | 1 |  |  |  |  |
| Insufficient support of the Executive Board for the project | 5 | 4 |  |  | 1 |  |
| Insufficient integration of quality improvement | 3 | 1 |  |  | 2 |  |
| Negative improvement culture | 1 |  | 1 |  |  |  |
| Insufficient available time | 13 | 8 | 4 | 1 |  |  |
| No room for training and education in quality improvement | 2 |  | 1 | 1 |  |  |
| Insufficient rewards and recognition for improving quality | 1 |  |  | 1 |  |  |
| Opponents of the project | 3 | 1 | 1 |  |  | 1 |
| Insufficient financial support for QIP | 1 |  | 1 |  |  |  |
| Data infrastructure (systems to use and pull data for QIP) | 6 | 1 | 2 | 2 |  | 1 |
| Division of labor | 1 |  |  | 1 |  |  |
| Other organizational changes (reorganization, merger) | 3 |  | 2 |  |  | 1 |
| Project plan not established in advance due to job change^a^ | 1 | 1 |  |  |  |  |
| No research structure^a^ | 1 |  |  |  | 1 |  |
| Turnover of residents^a^ | 1 |  | 1 |  |  |  |
| Organizational legislation and rules^a^ | 1 |  |  |  | 1 |  |
| No Salzburg signing^a^ | 1 |  |  | 1 |  |  |
| Logistical problems^a^ | 1 |  | 1 |  |  |  |
| Not invented here^a^ | 1 |  |  |  | 1 |  |
| Too much other QIPs^a^ | 1 | 1 |  |  |  |  |
| Management reviews in English^a^ | 1 |  |  | 1 |  |  |
| Not enough resources available^a^ | 1 |  | 1 |  |  |  |
| Different locations^a^ | 1 | 1 |  |  |  |  |
| **External environment (N=7)** |  |  |  |  |  |  |
| Legislation | 1 |  | 1 |  |  |  |
| Pressures or incentives | 2 |  |  | 1 | 1 |  |
| Involving stakeholder too late^a^ | 1 |  |  | 1 |  |  |
| Paperwork and accountability master program^a^ | 1 | 1 |  |  |  |  |
| Lack of support by approval QIP Committee of Research involving Human Subjects^a^ | 1 |  |  | 1 |  |  |

^a^ Self-experienced barriers by professionals (not included in this way in the pre-specified list of barriers used in the survey)

^b^ Number of times a barrier was ranked in a top 5

^c^ Number of times a barrier was ranked on a specific place in the top 5
