# Supplementary material for: Development of a tool for identifying and addressing prioritised determinants of quality improvement initiatives led by healthcare professionals: a mixed-methods study
Source: Implement Sci Commun. 2020 Oct 23;1:92. doi: 10.1186/s43058-020-00082-w (PMC7584081; doi:10.1186/s43058-020-00082-w)
Supplement: Supplementary file 5 — Additional file 5. Tool determinants QI initiatives. [file 43058_2020_82_MOESM5_ESM.docx]

**Additional file 5 . Tool with barriers and facilitators for performing QI initiatives**

| Hindering determinants | Target population | Analysis (diagnosis) | Range of  impact or  involvement^1^ | Approaches  (how do I make sure the QI initiative is hindered as little as possible by these determinants) |
| --- | --- | --- | --- | --- |
| *Intervention level* |  |  |  |  |
| Lack of evidence in literature of the effects of intervention (24^2^) | Intervention in the QI initiative | - Using the ladder of evidence, see which research design was employed to investigate the effect of the intervention and what the effect itself was | Impact | - Look for a systematic review of the intervention - Conduct a rapid review of the intervention - Conduct an effects synthesis - Translate the effect to the unique context by comparing differences between the unique context and the context of evidence for intervention |
| *Department level* |  |  |  |  |
| Insufficient motivation among the workforce (21^2^) | Employees who have to work with the intervention | - Do employees show commitment to the QI initiative , e.g. by being present at meetings, honouring agreements, and having the QI initiative discussed during work meetings? - Are employees saying that they are not motivated to work on the QI initiative? | Impact | - Make the employee take ownership of the problem, e.g. by giving them practical examples or letting them experience the problem from their own perspective - Clearly show that the QI initiative has been integrated with other issues in the department by making it clear how the project fits in with other ongoing projects or things happening in the department - Open the personal interests and underlying reasons for employees' lack of motivation up for discussion by approaching them individually. A facilitating approach is helpful: ask questions such as “what can I do for you in order to convince you to participate in this initiative ”? - Proactively inform department management about the QI initiative and ask them to bring the initiative t to the attention, such as by sending the newsletter or addressing it at the start of the day |
| Experiencing one’s competencies needed for the intervention as insufficient (20^2^) | Employees who have to work with the intervention | - Have employees fill in a number of questions from the Dutch General Self-efficacy Scale (or another self-efficacy instrument), adapted to the intervention in the initiative and analyse the answers with the QI team | Impact | - Provide appropriate training. The training format depends on the intervention but it must be safe. Options include bed-side teaching, coaching, observation, or demonstration - Think critically about where in the initiative employees can learn about the new intervention. Also consider informal learning, such as by talking to colleagues. - A facilitating approach is helpful: ask employees what kind of training will help them to feel competent in working with the intervention - Enable employees to understand why they are working with the intervention and why this is the new framework for behaviour. Joint critical reflection with the team about their experience working with the intervention may help here - Provide explicit feedback to employees about their actions in any form of training |
| *Organisation level* |  |  |  |  |
| Insufficient available time (767^2^) | Project manager | - Evaluate whether you are on schedule during meetings with the QI team. If not, why is this the case and what could have caused it? | Impact | - Demonstrate leadership by discussing the cause of the perceived insufficient available time with the stakeholder, depending on the cause |
| Insufficient support of the Executive Board for the project (110^2^) | Executive Board or those tasked with the quality portfolio in the organization | - Conduct a stakeholder analysis - Has the QI initiative plan been signed by a member of the Executive Board? | Impact | - Make sure your initiative fits the mission or the strategic principles of the Executive Board and show this explicitly in the title of your initiative - Take someone else from your QI team to a meeting with the Executive Board so that you can complement each other |
| Insufficient integration of quality improvement (27^2^) | Hospital | - Is the improvement of the quality of care part of the mission/strategic principles of the hospital? - What is the contact about the initiative with the Executive Board like? - What is the response to the initiative ? (E.g. via a *stafconvent* (staff assembly)) - Is the institution accredited? - Fill in a questionnaire about the learning/safety culture, such as the COMPaZ, and discuss it with your QI team | Involvement | - Adjust your own expectations about embedding and disseminating your QI initiative; this will be difficult. First, put energy into implementing the initiative in your own department |
| Opponents of the project (30^2^) | Opponents in the hospital | - Identify what the consequences of the initiative are for other disciplines and departments using a stakeholder analysis - If the stakeholders are important for the implementation of the initiative , review how they look at the project by, such as by inviting them to an information session or by talking to them. Listen carefully and take any resistance seriously | Impact | - Take resistance seriously and try to find a common denominator, e.g. the benefits from the patient's perspective - In extreme cases and if there is the possibility of doing so, reposition your opponents. Demonstrate leadership by talking to the person who is in a position to take these steps. If you are in this position yourself, you can talk to your opponents about it |
| Data infrastructure (120^2^) | Data needed to measures the outcomes of the initiative | - Think about what you want to know and measure by formulating an outcome measurement - Consider whether the required data has already been registered as part of the healthcare process - If so, ask yourself where, by whom, and how the data is being registered and whether it can be extracted for the initiative. - If not, ask yourself how you can still obtain the data, such as by conducting questionnaires or making observations. | Impact | - Align your outcome measurement with data that has already been recorded as part of the healthcare process, preferably digitally - Seek advice from an epidemiologist or statistician - Involve someone from ICT for the collection of the data - Request support when using data management systems - Check with other departments about how they deal with the desired data registration - Be creative; if it is not possible to obtain the desired data, come up with other relevant outcome measurements that can be identified |
| Other organisational changes (reorganisation, merger) (27^2^) | Hospital | - Conduct a Strengths, Weaknesses, Opportunities, Threats (SWOT) analysis and discuss with your QI team the extent to which the initiative can be (negatively) influenced by the “threats” | Involvement | - If possible, adapt the intervention to the change - Accept that the initiative may be delayed - Find other project managers and discuss how they deal with changes in their initiative. Join forces by addressing issues together, such as reporting on the new situation |

^QI indicates quality improvement^

^1 The circle of impact contains the elements/people/contexts that you can have an influence on. The circle of involvement contains elements/people/contexts that you are involved by but that you do not have any influence on or where it is difficult to influence things.^

^2^  ^The priority score of the determinant. This priorty score consisted of the sum of the ranking in the top five (i.e., a determinant ranked in first place scored five points, a determinant ranked fifth place scored one point), multiplied by the number of times a determinant was placed in a top five by professionals. Determinants with a priority score ≥ 20 were included in our tool.^

| Facilitating determinants | Target population | Analysis (diagnosis) | Range of  impact or  involvement^1^ | Implementation (how do I make sure this determinant is present in my QI initiative) |
| --- | --- | --- | --- | --- |
| *Intervention level* |  |  |  |  |
| Intervention fits in with  the current workflow (120^2^) | Workflow improvement context | - Together with your QI team, describe the process of the intervention. Describe the process of the current situation with regard to healthcare in your own context. Analyse the gap between the process description of your own context and that of the intervention process | Impact | - Make the intervention appropriate for application within the current process description of your own context. Describe what the desired care is in your own context at the process level - When there is more experience in leading improvement initiatives, initiatives can also be used adaptively. In these initiatives there is room to make mistakes and to experiment |
| *QI team level* |  |  |  |  |
| Sufficient participation in  the decision-making process by team members (24^2^) | team members of the QI initiative | - Have your team members fill in the participation questions of the Team Climate Inventory, and discuss this - Conduct a Strengths, Weaknesses, Opportunities, Threats (SWOT) analysis with your team - Ask team members to indicate, on a scale of 1 to 10, the extent to which they feel that decisions about the initiative are made on the basis of the input or opinion of the team member | Impact | - Discuss the results of the SWOT analysis with each other and decide who will take on which activity. Give the project member ownership of this activity - Make sure there are experts in your QI team and give them the floor during meetings by asking them for their opinion and/or experience - Create a dialogue in which discussion is avoided. Ask clear questions, listen carefully, and leave room for conversation. Come up with a plan by exploring - At the start of the team meeting, indicate that you have a role as a process supervisor in which you actively ask for everyone's opinion and ensure that everyone has the ability to provide input |
| *Department level* |  |  |  |  |
| Sufficient support of  management (288^2^) | Department management | - Conduct a stakeholder analysis - Does management meet its agreements? Are you involved in matters relating to the initiative? Do you receive information about the initiative? - Are a member of the Executive Board and a department head the project sponsor? Do they actively support it and provide resources? | Impact | - Seek support from management before the start of the initiative - At least once every 9 months, according to an agreement with the management, provide periodic information about the progress of the initiative. This is part of a detailed communication plan - Explicitly ask the department head and a member of the Executive Board to sign the project plan |
| Employee support (203^2^) | Employees who will be working with interventions | - The initiative is reflected in the annual planning of the department - The initiative is an item on the agenda at official meetings - Conduct a support analysis as part of a stakeholder analysis | Impact | - Include key members of the department in your improvement team, such as the department head and a patient - State what the problem is in a clear message. Formulate this from a workplace perspective (e.g. from the patient's perspective) - State what the initiative entails in a clear message. Formulate this from a workplace perspective - State what the initiative provides in a clear message. Formulate this from the perspective of the workforce - Increase employee ownership by ensuring that employees are given the freedom to respond to the initiative, e.g. by creating an anonymous comment/suggestion box - Be open to comments and suggestions for the initiative by asking questions: “What do you think?”/“Do you have any alternative ideas?” |
| Bottom-up project approach (96^2^) | The workplace | - Complete a partnership map with the primary stakeholders of the initiative. This describes everyone's motive for collaborating on the initiative. Engage in discussion about this - Ask yourself where the initiative idea comes from. Is it the Executive Board, you, or the workforce? | Impact | - Make sufficient contact with all levels of the organisation based on a communication plan, especially with key members who were identified in the stakeholder analysis - Put together a sounding board group (six to eight people) consisting of different people from the department. Periodically inform them about the progress, barriers, and results of the initiative and have them brainstorm with you - Include key members of the department, who were identified in the stakeholder analysis, in your QIteam - Be visible in the workplace - Make sure the workforce also experiences it as a problem and ask this to them during the first meeting about the annual plan |
| Enthusiastic/motivated department head (76^2^) | Department head | - Does the department head does what he/she says he/she does/will do? - Does the head of department show visible commitment, such as by satisfying agreements, supplying resources, and providing information? - Ask the department head directly if they are enthusiastic about the initiative and ask them why | Impact | - Make the department head a member of the QI team - Maintain proactive personal contact both formally and informally about the progress of the initiative |
| The workforce is motivated about the improvement project (33^2^) | The Workplace | - Include one or two employees in your QI team and ask them regularly what is going on in the department - Organise a meeting with the workforce in which attention is paid to what is going on in the department and what people think of the initiative. Have employees provide arguments for whether or not they are motivated - Audit the department and see 1) how employees react to the initiative; indifferent or passionate? 2) Are employees in active mode or in passive mode? 3) Do employees do what has been agreed on? | Impact | - Take motivation seriously - Use motivated employees as role models to motivate others by allowing them to help others with the implementation and try things out first - Show the problem you are tackling explicitly, not only through data but also through emotion. For example, bring someone along who has had to deal with the problem you are addressing in your initiative - Motivate employees extrinsically when intrinsic motivation is absent. This may be intangible (e.g. complementing them) or material (e.g. salary increase) - Show that the solution also fixes the problem. You can do this using logic or on the basis of literature - Exhibit enthusiasm in the workplace and within the QI team by showing them that the initiative yields something |
| *Organisation level* |  |  |  |  |
| Culture of improvement (60^2^) | Hospital | - Are people open to each other about what goes well and what doesn't? Do they do anything about it? - Discuss with your improvement team the scales for “non-punitive response to mistakes”, “open communication”, and “feedback and learning from mistakes” from the COMPaZ or another measuring instrument about safety culture - Is there a data infrastructure from outcome data on quality of care? | Involvement | - Set an example as an employee within a learning culture, such as by asking feedback to others about the initiative, and be transparent by sharing outcomes with stakeholders |
| Sufficient available time (90^2^) | Project manager | - Evaluate whether you are on schedule during meetings with the QI team. If not, why is this the case and what could have caused it? | Impact | - Show leadership by discussing the cause of the perceived sufficient available time with the stakeholder, depending on the cause |
| Sense of urgency (42^2^) | Executive Board or those tasked with the quality portfolio | - Can stakeholders themselves indicate how the initiative contributes to the objectives or vision of the organisation? - Is the initiative visible in the agenda of meetings? - Do you successfully make agreements? Are stakeholders present at meetings? - Will funds be made available for the implementation of the initiative? | Impact | - Ask yourself and the QI team ‘five times why’. Communicate these answers to the Executive Board. - Adapt your initiative goal to one that suits the hospital's strategic principles at that time - Stimulate the sense of urgency from the perspective of the Executive Board. For example, use the Triple Aims |
| Sufficient support of expertise in the field of quality improvement (95^2^) | Experts within the organisation | - Get to the heart of the problem so you know the area in which you require expertise - Take a look at the composition of your QI team: the number of people, men/women, age, and expertise. Look at the variety and level of expertise. Try to connect this as much as possible to what needs to be done. Then find out if the expertise in your QI team is lacking | Impact | - Make it clearly visible that you have brought in and involved certain expertise. This will give your initiative more credibility - At the start of the initiative, think about what expertise you might need - Bring someone with you from the start of the initiative who makes the urgency and purpose of the initiative clear. Ask them about the commitment to support the initiative when needed - Include the most important expertise in your QIteam. Involve other expertise expertise periodically when needed. When delivering the initiative, make sure that these persons also receives due credit. Make good agreements on this - Provide the resources and commitment to use the expertise - Use your social network to use expertise. This help is reciprocal in nature |
| *External environment level* |  |  |  |  |
| Incentives or pressure (financial, legal, or political) (64^2^) | External environment | - Conduct a Strengths, Weaknesses, Opportunities, Threats (SWOT) analysis with your QI team and focus on the “Opportunities” (for example, attention to the subject at IGJ (healthcare inspectorate)) | Involvement | - As a project leader, you have no influence on the presence or absence of external pressure or incentives - Try to connect to subjects where “pressure” exists or frame your initiative so that it fits with these |

^QI indicates quality improvement^

^1 The circle of impact contains the elements/people/contexts that you can have an influence on. The circle of involvement contains elements/people/contexts that you are involved by but that you do not have any influence on or where it is difficult to influence things.^

^2^  ^The priority score of the determinant. This^ ^priority score consisted of the sum of the ranking in the top five (i.e., a determinant ranked in first place scored five points, a determinant ranked fifth place scored one point), multiplied by the number of times a determinant was placed in a top five by professionals. Determinants with a priority score ≥ 20 were included in our tool.^
